# Genome-wide DNase hypersensitivity, and occupancy of RUNX2 and CTCF reveal a highly dynamic gene regulome during MC3T3 pre-osteoblast differentiation

**DOI:** 10.1371/journal.pone.0188056

**Published:** 2017-11-27

**Authors:** Phillip W. L. Tai, Hai Wu, André J. van Wijnen, Gary S. Stein, Janet L. Stein, Jane B. Lian

**Affiliations:** 1 Department of Biochemistry, University of Vermont College of Medicine, Burlington, Vermont, United States of America; 2 Mayo Clinic, Rochester, Minnesota, United States of America; Kyungpook National University School of Medicine, REPUBLIC OF KOREA

## Abstract

The ability to discover regulatory sequences that control bone-related genes during development has been greatly improved by massively parallel sequencing methodologies. To expand our understanding of *cis*-regulatory regions critical to the control of gene expression during osteoblastogenesis, we probed the presence of open chromatin states across the osteoblast genome using global DNase hypersensitivity (DHS) mapping. Our profiling of MC3T3 mouse pre-osteoblasts during differentiation has identified more than 224,000 unique DHS sites. Approximately 65% of these sites are dynamic during temporal stages of osteoblastogenesis, and a majority of them are located within non-promoter (intergenic and intronic) regions. Nearly half of all DHS sites (both constitutive and dynamic) overlap binding events of the bone-essential RUNX2 and/or the chromatin-related CTCF transcription factors. This finding reinforces the role of these regulatory proteins as essential components of the bone gene regulome. We observe a reduction in chromatin accessibility throughout the genome between pre-osteoblast and early osteoblasts. Our analysis also defined a class of differentially expressed genes that harbor DHS peaks centered within 1 kb downstream of transcriptional end sites (TES). These DHSs at the 3’-flanks of genes exhibit dynamic changes during differentiation that may impact regulation of the osteoblast genome. Taken together, the distribution of DHS regions within non-promoter locations harboring osteoblast and chromatin related transcription factor binding motifs, reflect novel cis-regulatory requirements to support temporal gene expression in differentiating osteoblasts.

## Introduction

The process of osteoblast differentiation is controlled by an abundance of cellular signaling events that impact the regulation of gene transcription, and in turn, direct cellular identity and behavior. Key transcription factors such as RUNX2, osterix (Sp7), ATF4, homeobox proteins, AP-1 factors, and hormone receptors regulate the bone program [1, 2]. Knockout and overexpression of these factors have revealed their critical roles in bone formation, and extensive promoter analyses of individual bone-essential genes have shown how these factors can directly bind DNA motifs to activate or repress gene transcription. The extent to which these factors globally contact the genome has been made possible by high-throughput strategies, leading to the recognition that gene regulation extends far beyond promoter regions [3, 4]. Although studies are decoding how genes are activated, repressed, poised, and interact with each other in 3-dimensional space by epigenetic regulators of chromatin [5, 6], current knowledge and approaches have only defined a fraction of the osteoblastic regulome. Only a few studies have directly looked at the global contribution of transcription factors in a differentiation model for bone formation [7–10].

DNase I hypersensitivity is an unbiased approach that reveals chromatin regions accessible to nuclease activity due to displacement or depletion of nucleosomes caused by the binding of transcription factors or factor complexes [10, 11]. Thus, DNase hypersensitivity (DHS) is a powerful identifier of active *cis*-regulatory regions [11, 12]. For example, the ability to define sequence regions that are responsive to bone-related cues, such as Runx2 [7, 8, 13], the Dlx family of factors [14], and Vitamin D induction [6, 15–18], has illustrated the usefulness of DHS analysis to characterize transcriptional activity during osteoblast differentiation. When scaled to examine global DNase hypersensitivity via DNase-seq [19], the genome-wide characterization of all active *cis*-regulatory regions throughout a differentiation program is possible.

In this study, three hallmark differentiation stages of MC3T3 osteoprogenitor cells [20] were profiled by DNase-seq analyses. Our analysis shows that strong DHSs at promoters represent only 10% of all DHS. Although, many of these promoter DHSs are associated with bone-related genes that are expressed in mature osteoblasts, highly dynamic changes in chromatin accessibility were found largely at intergenic and intronic sequences at all stages of osteoblast differentiation. Furthermore, nearly 50% of all DHS regions in differentiating osteoblasts are targeted by the Runt-related transcription factor 2 (RUNX2) and/or the CCCTC-binding factor (CTCF). Our results highlight their essential roles in the osteoblast differentiation program [1, 21] and chromatin organization [22] by revealing their association to such a large percentage of the entire osteoblast *cis*-regulome. In addition, we report the discovery of DHS sites downstream of transcriptional end sites (TES), and identify of a class of genes associated with these nuclease-accessible regions that indicate a novel mode of gene control. This specific category of genes is related to more than 10 major pathways that reflect bone development and signal transduction processes, including genes not previously linked to the bone differentiation program. Importantly, our data significantly contribute to the growing resource of identified global DHS sites at distinct stages of osteoblastogenesis. Our unique interrogation of 3’-DHSs during osteoblast differentiation has revealed a novel mode of regulation that may also be occurring in other differentiation models.

## Materials and methods

### Cell culture

The MC3T3-E1 clone-4 pre-osteoblastic murine cell line [20] (American Type Culture Collection, Manassas, VA) was used in this study. Growth-phase cultures were maintained as reported previously [7, 23]. When cultures reached ~90% confluency, differentiation was initiated by the addition of 142 μM ascorbic acid (Sigma-Aldrich, St. Louis, MO) to 10% FBS (Hyclone, Thermo Fisher Scientific) in α-MEM. After 2 days, the ascorbic acid concentration was increased to 280 μM, and 5 mM β-Glycerophosphate (Sigma- Aldrich) was added [20]. Cultures were maintained at 37°C at 5% CO_2_, with fresh media changes every 2 days. Phase contrast images of differentiation progression were captured on a Nikon Eclipse TS100 inverted microscope (Nikon Instruments Inc., Melville, NY), conjugated to a SPOT RT3 CCD camera using SPOT imaging software v5.0 (Diagnostic Instruments Inc., Sterling Heights, MI).

**Fig 1:**
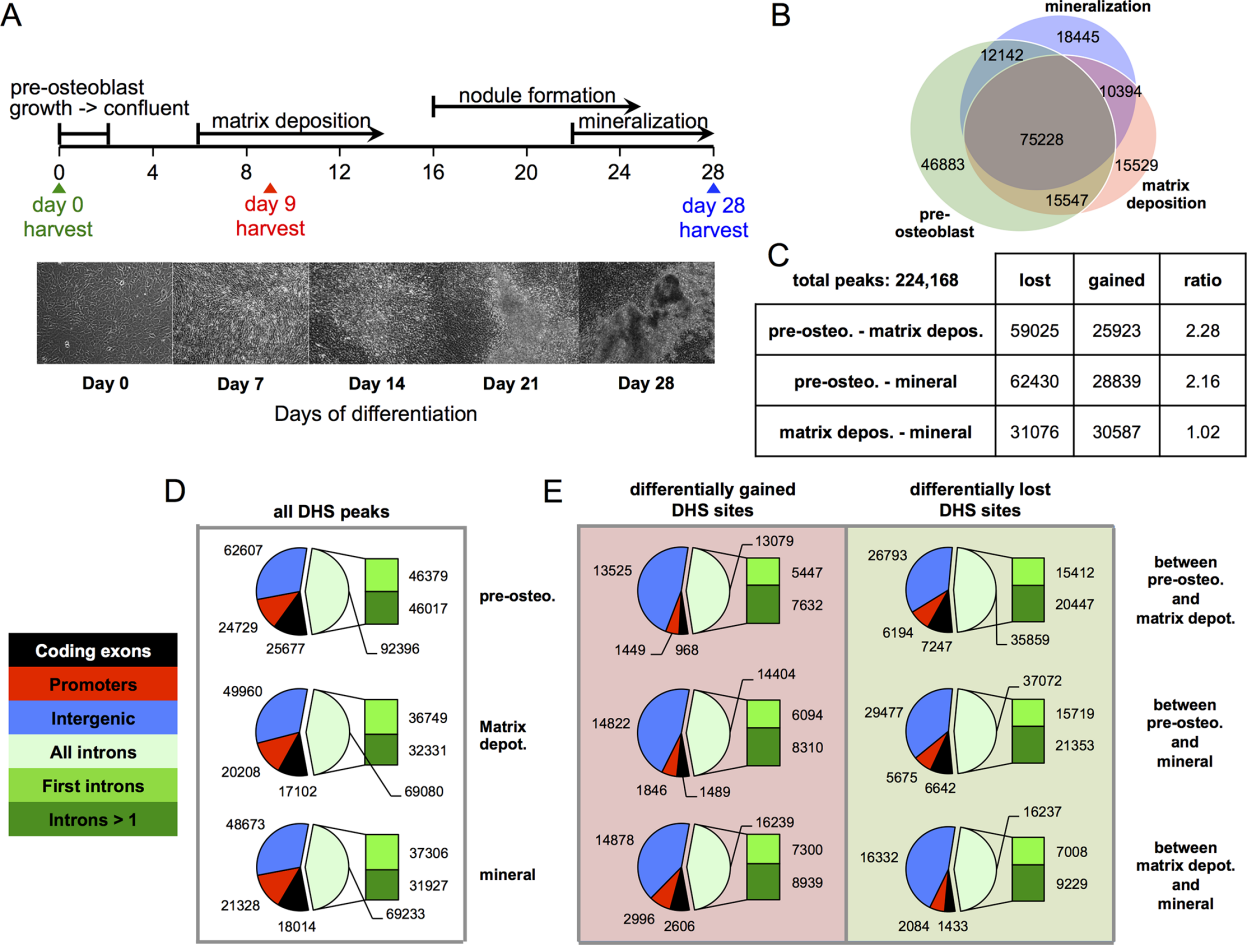
Differentiating mouse MC3T3-E1 osteoblasts are marked by dynamic gain and loss of DHS sites. **(A)** Three hallmark stages of osteoblast differentiation were collected for DNase-seq library builds (pre-osteoblasts, matrix deposition, and mineralization). Phase images of differentiating MC3T3-E1 cultures captured every 7 days post-switching to differentiation conditions. Each panel illustrates that differentiating cultures follow the predicted nodule formation indices between days 14 and 21 and heavy mineralization by day 28. **(B)** Venn diagram illustrating the overlaps of DHS sites at the three osteoblast stages. Values accompanying diagram portions reflect absolute peak counts. **(C)** A table of peak numbers lost or gained and their ratios between each stage of osteogenesis. **(D)** Pie charts of DHS site distributions at pre-osteoblasts, matrix depositing osteoblasts, and mineralizing osteoblasts. The distribution of DHS peaks among genomic partitions: coding exons (black), promoters (red), intergenic sequence (blue), and intronic sequence (green) are not mutually exclusive. **(E)** Pie charts of DHS sites that are differentially gained (left column), and differentially lost (right column) are shown. Values accompanying diagram portions reflect absolute peak counts.

MC3T3-E1 cultures were differentiated for a period of 28 days and cells representing the three hallmark stages of differentiation: proliferation (day 0), matrix deposition (day 9), and mineralization (day 28) were collected (see below). The differentiation kinetics of parallel cultures was verified by qPCR analysis (S1 Fig), visualization of nodule formation (multi-layering of mature osteoblasts), and matrix mineralization (Fig 1A) [7, 23]. Standard Alkaline phosphatase activity, Alizarin red, and Von Kossa staining of cultures were also performed as previously described [7, 10].

### RT-qPCR

Total RNA from cultures was extracted with TRIzol (Invitrogen, Life Technologies, Grand Island, NY), followed by DNase treatment using the DNA-Free RNA Kit (Zymo Research, Irvine, CA) according to manufacturers’ instructions. cDNA was prepared using the SuperScriptIII First-Strand Synthesis System (Invitrogen). qPCR was performed with the iTaq SYBR Green Supermix with ROX (Bio-Rad, Hercules, CA) on the ViiA 7 Real Time PCR System (Applied Biosystems, Life Technologies, Grand Island, NY). Relative transcript levels were determined by the ∆∆*Ct* method, normalized to *gapdh*. Primer sequences for *runx2-P1*, bone gamma-carboxyglutamic acid-containing protein (*bglap2*), integrin-binding sialoprotein (*ibsp*), and glyceraldehyde 3-phosphate dehydrogenase (*gapdh*) are described elsewhere [24]. Additional primer sequences are provided in S1 Table and were designed using FoxPrimer (www.foxprimer.org; Dobson *et al*.).

### DNaseI treatment and massively parallel sequencing

Approximately 4 X 10^7^ growth-phase (day 0), matrix-deposition stage (day 9), or mineral stage (day 28) MC3T3-E1 clone-4 cells were harvested and subjected to DNaseI digestion according to methods described in Barutcu *et al*. 2014 [10]. Biological replicates 1 were sequenced by single-end 36-bp reads on an Illumina Genome Analyzer II platform at The University of Massachusetts Medical School Deep Sequencing Core Facility (Worcester, MA). Biological replicates 2 were sequenced by single-end 100-bp reads at the University of Vermont Advanced Genome Technologies Core on an Illumina Hiseq1000 platform. The increased sequencing depth achievable on the Hiseq1000 platform allowed for biological replicate 2 libraries to be processed by multiplexed sequencing, yielding approximately equivalent read depths for individually barcoded libraries on the GAII platform with single libraries per lane. Sequences of the 3’-barcoded adaptors were provided by Dr. Song and Dr. Crawford (Department of Pediatrics, Division of Medical Genetics, Duke University, Durham, NC) and are:

Multiplex1 5’ P-AGTCGTGATGTTCGTATGCCGTCTTCTGCTTG
Multiplex2 5’ P-AGTACATCGGTTCGTATGCCGTCTTCTGCTTG
Multiplex4 5’ P-AGTTGGTCAGTTCGTATGCCGTCTTCTGCTTG

Base calls were performed using CASAVA version 1.6. DNase-seq reads were aligned to the mm9 genome assembly using Bowtie (version 1.1.2) allowing up to two base mismatches. DNase-seq analyses were confirmed by two biological replicates, each constituting technical duplicates on significant peaks called by F-seq [25] using a standard deviation threshold value of 4. Normalized signal tracks were generated using align2rawsignal (Kundaje A., http://code.google.com/p/align2rawsignal/) on combined BAM files of technical replicates and displayed as bigwig tracks on the UCSC Genome Browser [26, 27]. DNase-seq tracks presented here have been deposited into the Gene Expression Omnibus (GEO) database (GSE55046). For brevity, tracks displayed in the manuscript represent DNase-seq tracks of biological replicate 2 only. S2 Table summarizes the total number of mapped reads and F-seq called peaks from biological replicates 1 and 2.

### Analyses of DNase-seq data

We employed a conservative selection of F-seq called DNase-seq peaks by only selecting peaks that were present in both biological replicates (common peaks) for each MC3T3-E1 stage analyzed. Therefore, unique peaks between each biological replicate, regardless of individual biological replicate peak strength, were considered false-positive calls and were discarded from downstream analyses. Common peaks were annotated as BED files and reported as DNase hypersensitive sites (DHS sites). Using this method, we consider that the DHS sites reported in this study to be high-confidence sites, since we have only reported hypersensitive regions that span called peaks present in both biological samples produced from two independent sequencing facilities, using different library build schemes (see above). As a result, ~40–50% of biological replicate 1 peaks overlap with biological replicate 2 peaks between each of the differentiation stages reported. Furthermore, the number of common DHS sites identified is within the observed range (between 110,000 and 150,000 peaks) in various cell lines by others employing similar methodologies [19].

DNase-seq bioinformatic pipelines were partially performed using tool sets available on the Galaxy web-based platform for genome data analysis [28–30], unless otherwise stated. Venn diagrams were drawn using eulerAPE [31]. Motif analysis was performed using the Hypergeometric Optimization of Motif EnRichment (HOMER) tool suite (version 4) [32] for *de novo* discovery of overrepresented motifs within DHS sites. Background sequences used to compare against DHS sites were generated automatically by HOMER. The calculated DHS length averages where used as the background sequence lengths. Genomic partitions (pie charts) were based on Ensembl gene predictions (archive data version 65 for NCBI37/mm9 assembly) [33]. Since larger DHS sites can span several genomic partitions, many DHS sites were tabulated several times in a non-mutually exclusive manner. Violin plots were made with the R package ggplotviolin.R (http://docs.ggplot2.org/0.9.3/geom_violin.html) in the RStudio environment (RStudio, Boston, MA). Aggregation plots and heatmaps were generated using ngs.plot (version 2.41) [34] using only combined mapped reads from both biological replicates that overlapped with common peaks (removes false-positive signals, and experimental noise). Aggregation plots and heatmaps cover either the gene body ±2 kb, or TES ±2 kb where applicable.

GO-term enrichment analysis was performed using the ClueGO module of Cytoscape [35, 36] using GO_BiologicalProcesses_20.3.2014_19h52 ontologies. Two-sided hypergeometric testing with Benjamini-Hochberg correction method was used. Term enrichment for both TES+1000 and TES+500 genes were defined by a minimum of 4 genes represented with a 2.0% minimum percentage coverage of genes within terms. For visualization purposes, cluster comparisons used a 51% percent association bias to establish gene cluster significance. GO-term connectivity (Kappa score) threshold = 0.5.

## Results

### Differentiating osteoblasts exhibit loss and gain of DNase hypersensitivity, especially within intergenic/intronic regions

To understand the global regulation of osteogenesis from the perspective of chromatin architecture, we examined differential nuclease hypersensitivity during the process of osteoblastogenesis by profiling the well-described mouse MC3T3-E1-clone 4 pre-osteoblast cell line [20]. MC3T3-E1 cells were differentiated for a period of 28 days (Fig 1A), and cells representing the three hallmark stages of differentiation were collected: proliferating pre-osteoblasts (d0), matrix depositing osteoblasts (d9), and mature mineralizing osteoblasts (d28). These samples were subjected to DNase-seq library builds and massively parallel sequencing analyses. We found that hypersensitive sites are highly dynamic among the three osteoblastic stages. A total of 224,168 unique DHS sites were observed (Fig 1B). DHS peaks that were common among all three stages totaled 75,228, while more than 65% (80,857 peaks) are exclusive to an individual osteoblast stage (pre-osteoblasts, 46,883; matrix depositing osteoblasts, 15,529; and mineralizing osteoblasts, 18,445) (Fig 1B). The predominant length of DHS regions is between ~100–500 bp with a range of 50 bp to >10 kb (S2A Fig).

We observed that more DHS sites were lost during differentiation than were gained. For example, between pre-osteoblasts and matrix depositing stages, 59,025 DHS sites were lost, while 25,923 were gained (Fig 1C). The DHS lost-to-gained ratio decreased between matrix depositing and mineralizing stages, with 31,076 peaks lost and 30,587 gained. The greater than two-fold loss-to-gain ratio between pre-osteoblasts and differentiating osteoblasts (matrix or mineralizing) suggests that proliferating pre-osteoblasts are characterized by a more accessible chromatin state that becomes more restricted during osteoblast commitment (Fig 1C). These findings show that open chromatin regions that define the multipotential mesenchymal precursor state are lost, while other DHSs are gained as differentiation progresses. These changes are likely indicative of *cis*-regulatory region silencing and activation that together regulate the osteoblast transcriptional profile during osteogenesis.

Because differentially hypersensitive regions are attributed to changes in osteoblast gene expression, we predicted that many of these DHS sites would be close to genes (within 10 kb), and associated with loss or gain of promoter or enhancer accessibility. We therefore examined DHS site positioning throughout the three hallmark osteoblast stages in relation to annotated gene bodies. DHS sites were categorized into four genomic partitions: coding-exons, promoters, intronic, and intergenic sequences (Fig 1D). Intronic sequences were further partitioned into first introns and introns beyond exon-2. As coding-exons and promoters can be embedded within intronic regions of syntenic genes and larger DHS regions can often encompass promoter, coding-exon, and intronic sequences simultaneously, many DHS sites were catalogued more than once for thoroughness.

We found that more than one-fourth of DHS sites are within intergenic sequences, while nearly half are within introns (Fig 1D). Of the sites present within introns, half are found in first introns. This distribution was consistent throughout the three osteoblastic stages. These observations are consistent with findings by others that show a high representation of DHS sites within the first introns and intergenic regions of genes in diverse cell and tissue types [12]. Notably, differential DHS sites identified within promoters tended to make up only a small percentage of all differential DHS sites (Fig 1E). DHS sites that are gained or lost between osteoblast stages are also largely found within intronic and intergenic regions. For example, 49,960 DHS sites are within intergenic sequence at the matrix depositing stage (Fig 1D). Of these, 13,525 (27.1%) are gained between pre-osteoblasts and matrix-depositing osteoblasts (Fig 1E, left panel), suggesting that more than a fourth of intergenic DHS sites are a result of gained DHS sites. Conversely, a total of 20,208 peaks were found within promoters, but only 1,449 peaks were differentially gained (7.2%). These trends are similar for differentially lost DHS sites, with the majority of these peaks being present within intronic and intergenic sequences (Fig 1E, right panel). Our findings indicate that dynamic changes occur predominantly within intergenic or intronic sequences and emphasize that dynamic DHS changes at promoters are dramatically underrepresented.

### DHS profiles reveal that RUNX2 and/or CTCF-centered transactivation contributes to nearly 50% of all regulatory events during osteoblastogenesis

**Fig 2:**
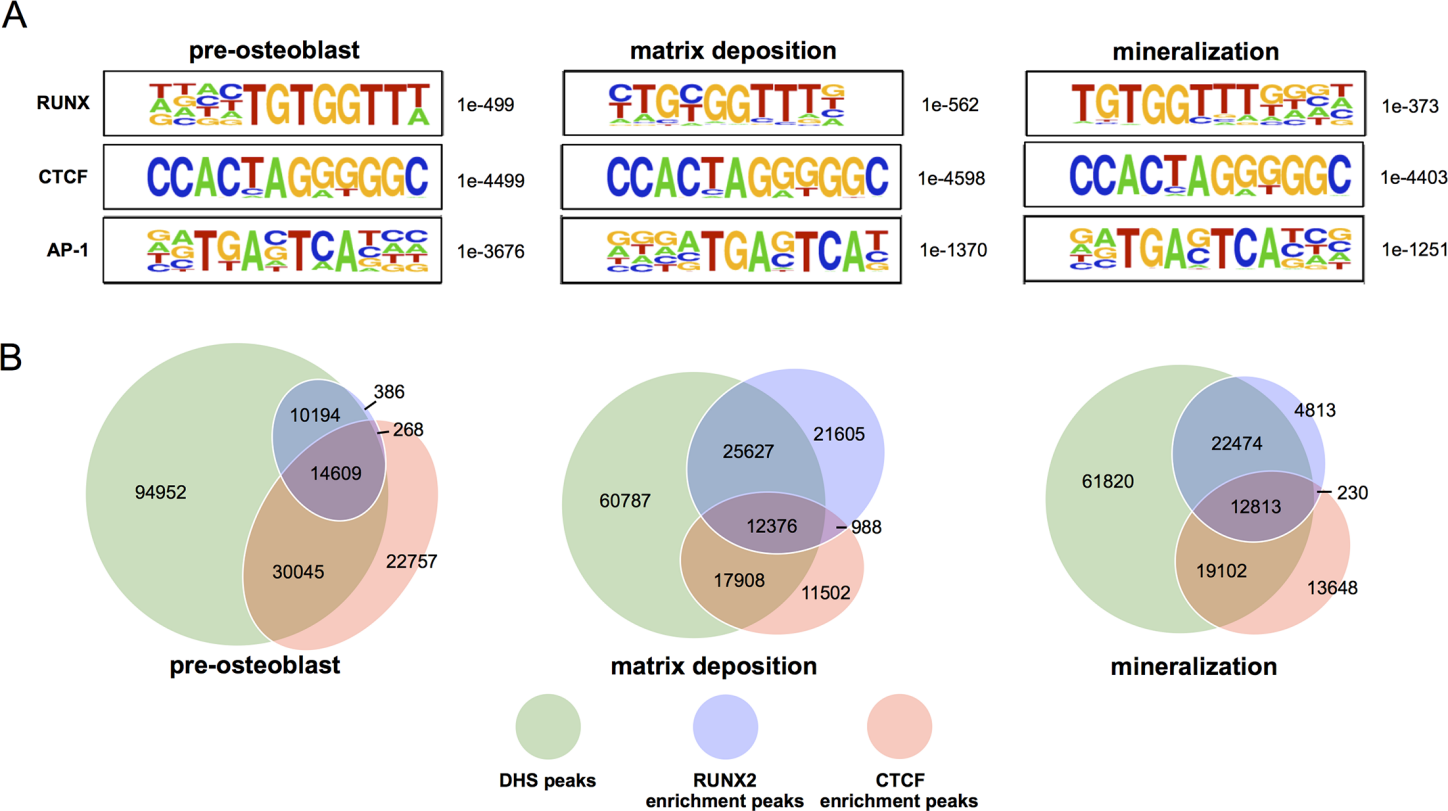
DHS sites highly overlap with RUNX2 and CTCF enriched regions. **(A)** HOMER analyses identifying *de novo* discovered motifs within DHS sites of pre-osteoblasts (left column), matrix depositing osteoblasts (middle column), and mineralizing osteoblasts (right column). Among the highest motifs enriched at regulatory regions throughout differentiation are RUNX (top row), CTCF (middle row), and AP-1 (bottom row). Significance of motif enrichments is represented by p-values listed to the right of each motif logo. **(B)** Venn diagrams of DHS sites (green), RUNX2 enriched peaks (blue), and CTCF enriched peaks (red) at the three hallmark osteoblast stages. Values accompanying diagram portions reflect absolute peak counts.

DHS sites are established by the binding of factors or factor complexes that displace or deplete nucleosomes, thus increasing nuclease accessibility [11]. We therefore reasoned that a bioinformatic analysis of motifs enriched within DHS sites would identify regulatory factors that establish the osteoblast transcriptional regulome. A large portion of DHS sites are within intergenic regions (Fig 1D) and may be related to enhancers that can interact with promoter sequences far-distal from gene bodies via looping interactions [37]. Thus, attributing relationships between discovered DHS and the closest gene by linear distance would be inaccurate. We therefore profiled all DHS positions to discover bone-related regulatory motifs at a global level. When DHS sites across osteoblast differentiation were subjected to sequence motif overrepresentation analysis using Hypergeometric Optimization of Motif EnRichment (HOMER) [32], CTCF (p < 1e-4400), RUNX (p < 1e-300), and AP-1 (p < 1e-1200) were among the top enriched motifs (Fig 2A). Detailed analyses of motifs in pre-osteoblasts, and at the matrix deposition and mineralization differentiation stages are shown in S3 Fig. The RUNX and AP-1 motifs, through the respective recruitment of RUNX2 and JUN/FOS complexes, are essential for the regulation of many known osteoblast genes [38–40]. Notably, we observed a change in the enriched RUNX motif (PyGPyGGTPy) at the matrix deposition stage, where the third core base is cytosine or thymine (5’-TG[C/T]GGTT-3’), whereas in pre-osteoblast or mineralization stages, the motif is strictly 5’-TGTGGTT-3’ (Fig 2A). The CTCF motif is associated with the recruitment of the zinc-finger CCCTC- binding factor, which is known for establishing chromatin architecture during development [3, 41]. Remarkably, the CTCF motif is well represented at all stages. (Fig 2A). Many of the motifs discovered (Sp1, TEAD, E2F1, Egr1, Nf1, Zfp161, Zfp281) are critical to the control of bone-related and general gene transcription [42–47] (S3 Fig). During matrix deposition, the motif for SMAD4, the essential regulator of TGFB and BMP signaling, is also enriched. Thus, enrichment of these sequence motifs demonstrates that the DHS sites identified in our study are consistent with the composition of known *cis*-regulatory regions of many key osteoblast-related genes [48].

Prompted by the overrepresentation of RUNX and CTCF motifs within DHS sites of differentiating osteoblasts, we next examined the extent to which regions of differential hypersensitivity coincided with RUNX2 and CTCF binding profiled by our previous ChIP-seq analyses [7]. We find that RUNX2 and/or CTCF enrichment events at DHS sites together make up nearly half of all DHS sites (Fig 2B). In fact, DHS sites that encompass either RUNX2 or CTCF enriched regions increased from 36.6% in pre-osteoblasts to 47.9% in matrix depositing osteoblasts, and to 46.8% in mineralizing osteoblasts (Fig 2B). More specifically, at the matrix deposition stage, RUNX2 is associated with 32.6% of all DHS sites while CTCF is associated with 26.0%. Notably, DHS sites that contain both RUNX2 and CTCF make up only 10% of all DHS accessible regions, suggesting that these two scaffolding factors can also function independently. Nonetheless, this finding indicates that these two factors together contribute to a large percentage of the osteoblast regulome.

Our analysis has also revealed that at peak levels of RUNX2 expression in matrix depositing osteoblasts [7], 37% of RUNX2 enriched regions do not overlap with DHS sites (Fig 2B), whereas in pre-osteoblasts and mineralizing osteoblasts, greater than 87% of RUNX2 enriched regions overlap with DHS sites. Therefore, this novel finding indicates that RUNX2 can associate with fully or partially inaccessible chromatin in a differentiation stage related manner, as the large percentage of non-overlap with DHS regions exists only in the matrix depositing stage. In contrast, the fraction of CTCF enriched regions present within DHS sites is consistent among the three osteoblast stages, with ~70% of CTCF enriched regions overlapping with DHS sites at all stages (Fig 2B).

**Fig 3:**
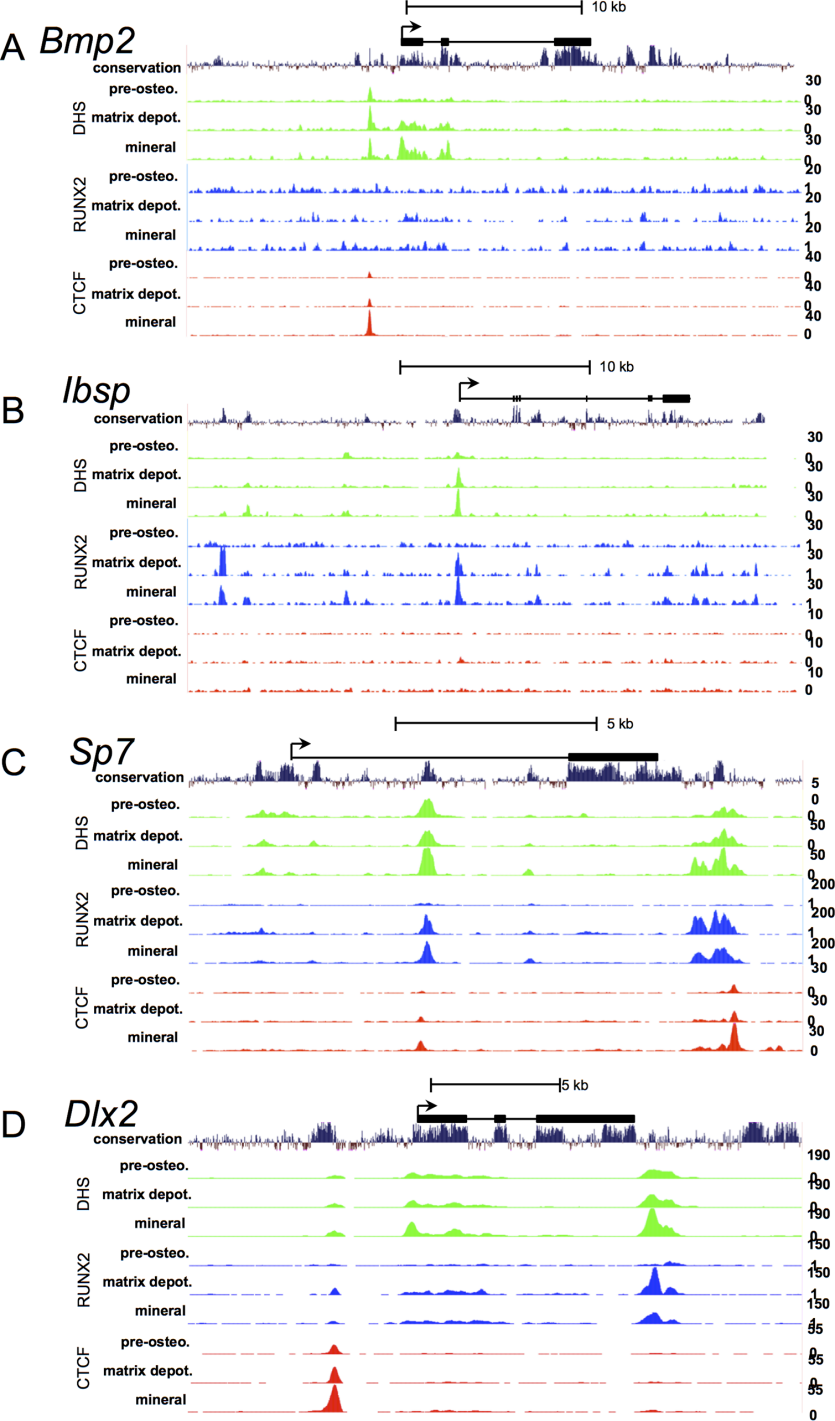
DHS, RUNX2, and CTCF enrichment tracks throughout differentiation of selected bone-related genes. Signal tracks of DNase-hypersensitivity (green), RUNX2 enrichment (blue), and CTCF enrichment (red) at pre-osteoblast, matrix depositing osteoblast, and mineralizing osteoblast stages; encompassing the bone-related genes **(A)** bone morphogenic protein 2 (*Bmp2*), **(B)** bone sialoprotein (*Ibsp*), **(C)** Osterix (*Sp7*), and **(D)** Distal-less homeobox-2 (*Dlx2*). The TSSs are designated by forward-facing arrows. 30-way Multiz Alignment and conservation tracks and diagrams of the gene transcripts accompany each track display above. The scales for the normalized signal values are displayed to the right of each track.

We next investigated whether bona fide bone-related genes exhibit dynamic nuclease accessibility proximal to their gene bodies during MC3T3 differentiation. DNase hypersensitivity was therefore examined at the *Bmp2 (bone morphogenic protein 2)*, *Ibsp (bone sialoprotein)*, *Sp7 (osterix)*, *and Dlx2 (distal-less homeobox-2)* genes during the hallmark osteoblast stages (Fig 3). Expression of the *Bmp2*, *Ibsp*, and *Sp7* are upregulated several-fold during differentiation in mature osteoblasts [49–51], while *Dlx2* is downregulated several-fold during early stages of osteoblastogenesis [52]. By cross-comparing RUNX2 and CTCF enrichments at these four gene loci, we observed different correlations between DHS locations and the enrichment dynamics of RUNX2 and CTCF (Fig 3). *Bmp2* exhibits increased CTCF binding during differentiation that is coincident with a DHS signal near the promoter (Fig 3A). *Ibsp* is strongly marked by RUNX2 enrichment and DHS signal at the TSS with no significant contribution by CTCF. A position upstream of the *Ibsp* promoter (-12.5 kb) exhibits strong enrichment of RUNX2 that does not correspond to a strong DHS peak at any of the tested osteoblast stages. This peak of DHS-free/RUNX2 enrichment (Fig 3B) is representative of the class of RUNX2 binding events that appears at the matrix-deposition stage but does not coincide with strong nuclease accessibility. This example and others throughout the genome, confirm that such events are not due to anomalies in bioinformatics analyses. For *Sp7*, all three modifications are present at an intronic region and near the transcription end site (TES) (Fig 2C). *Dlx2* is strongly enriched with CTCF upstream of the promoter, and by RUNX2 downstream of the TES.

We note that at the differentially expressed *Sp7* and *Dlx2* genes, DHS signals at the promoter sequences are relatively weak and change marginally between differentiation stages. However, there are striking differential DHS signals observed 3’ of the TES of these genes with the strongest DHS sites observed at mineralization stages. The *Sp7* gene exhibits differential DHS events spanning from +0.92 kb to +1.87 kb beyond the TES, and is defined by multiple peaks at the matrix deposition and mineralization stages (Fig 3C). The *Dlx2* gene exhibits dynamic DHS 3’ of the TES, centered at TES +300 (Fig 3D). In the case of *Sp7*, this regulatory region may behave as an enhancer, while in the case of the *Dlx2* gene the region may behave as a repressor. Both genes are required for differentiation to mature osteoblasts [50, 52]. Many of the TES DHS signals appear greatest at the mineralizing stage, suggesting that TES DHS is a unique feature of genes that are critically required for the terminal differentiated of osteoblasts. Further interrogation of the TES showed that there are no currently known transcripts originating from these sequences. Changes at these positions may therefore reflect the presence of dynamic regulatory regions present at the 3’ ends of these genes.

### A subclass of differentially expressed genes during osteoblastogenesis exhibit unique DNase hypersensitivity at the 3’-flank

**Fig 4:**
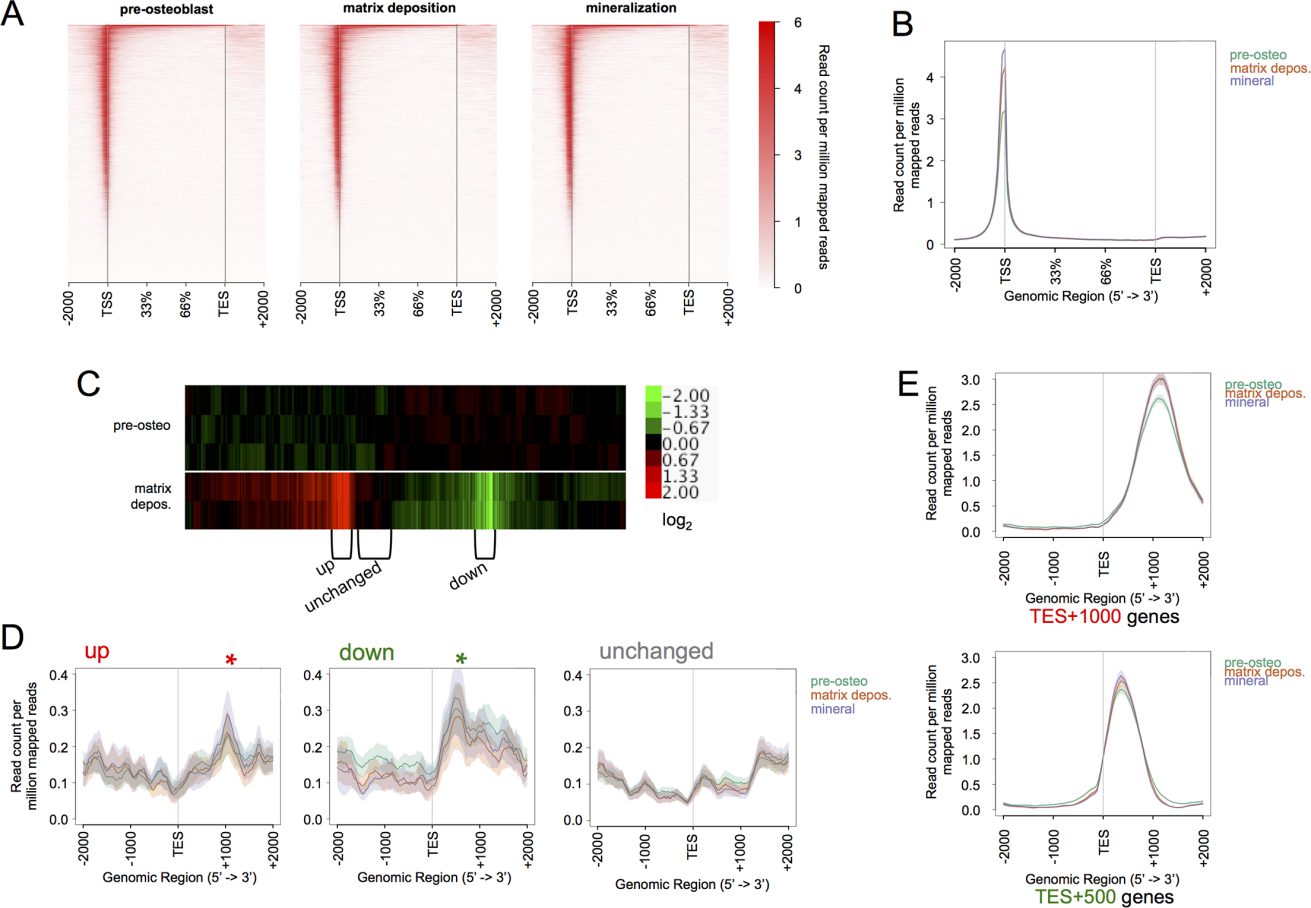
Regions proximal to the TSS exhibit the highest DHS signals, while regions 3’-flanking the TES display weaker but differential hypersensitivity signals. **(A)** Heatmap representation of genome-wide DNase hypersensitivity peak signals displayed at gene bodies ± 2 kb with quantile distribution from TSSs to transcriptional end sites (TESs). The relative signal intensities at annotated gene bodies are stacked along the y-axis. Note that there are a multitude of weaker peaks found within gene bodies that contrast with the strong signals from peaks at the TSS. **(B)** Aggregation plot display of heatmaps showing the average read counts per million mapped reads of genome-wide DHS signals for pre-osteoblasts (green), matrix depositing osteoblasts (red), and mineralizing osteoblasts (purple) cultures. Shaded areas represent ± SE. **(C)** Heatmap of Affymetrix data showing differentially expressed genes in MC3T3 cultures between pre-osteoblast (n = 3) and matrix depositing osteoblast (n = 2) stages [7]. The scale bar of the log_2_-fold change in expression is displayed to the right. The average transcript detections of individual genes at d0 are used as baseline (scaled to 0.00). Brackets designate node-selection of gene clusters that encompass those that are ≥2-fold upregulated, ≥2-fold downregulated, and unchanged. **(D)** Aggregation plots of DHS signals at the TES ±2kb of the 300 genes classified as upregulated (left plot), 282 genes classified as downregulated (center plot), and 371 classified genes shown to not significantly change (right plot) between pre-osteoblasts and matrix deposition stage. Asterisks indicate peak signal averages aggregating at TES+1000 sequences (red asterisk), or TES+500 sequences (green asterisk). **(E)** Aggregation plots of all TES+1000 (765 genes) and TES+500 genes (829 genes) spanning the TES ±2kb at pre-osteoblasts (green), matrix depositing osteoblasts (red), and mineralizing osteoblasts (purple).

We investigated whether the observations made of the *Sp7* and *Dlx2* genes represented a specific class of genes regulated by differential DHS at the 3’ ends of genes during osteoblastogenesis. Heat maps and signal aggregation of DHS peaks among the three hallmark stages of osteoblastogenesis were constructed to visualize the average prevalence of accessibility across gene bodies ±2 kb (Fig 4A and 4B). Hypersensitivity near genes was strongest at promoters (immediately 5’ of the TSS), but significant hypersensitivity was also observed within sequences 2 kb downstream of transcriptional end sites (Fig 4A and 4B). As DHSs at promoters show little change throughout differentiation (Fig 1E) and seem uniformly high among most genes, we proposed that the more numerous but differential DHS signals at 3’-ends of genes, could be informative of gene expression change.

To address whether specific 3’-DHS peak signal profiles correlate with differences in transcript levels during osteoblastogenesis, we used the set of genes differentially expressed during MC3T3-E1 differentiation that was characterized in a previous study [7]. Our analyses focused on genes expressed between proliferation and matrix deposition stages (Fig 4C and 4D). We identified three groups of genes: those that are upregulated (up), downregulated (down), and those that do not significantly change (unchanged) between day 0 and the early differentiation stage of day 9 (Figs 4C). These genes were defined by specific node groups generated by a two-way hierarchical clustering of expressed genes. The grouping sets encompass genes with two-fold change or more in expression between d0 and d9. Among these sets of expressed genes, we found that the average DHS signal intensities of upregulated genes have a characteristic peak that is centered ~1 kb downstream of the TES (Fig 4D, left plot), while the downregulated genes, were associated with a strong hypersensitivity signal peak centered near ~0.5 kb downstream (Fig 4D, center plot). The group of unchanged genes exhibited a lower and flatter distribution of reads within 1 kb downstream of the TES (Fig 4D, right plot). Based on these unique observations, we defined, two gene groups with hypersensitivity peaks centered near TES+1000 (765 genes) and TES+500 (829 genes). We did not observe significant differences in peak signals at the TES+1000 or TES+500 positions between differentiation stages (Fig 4E).

**Fig 5:**
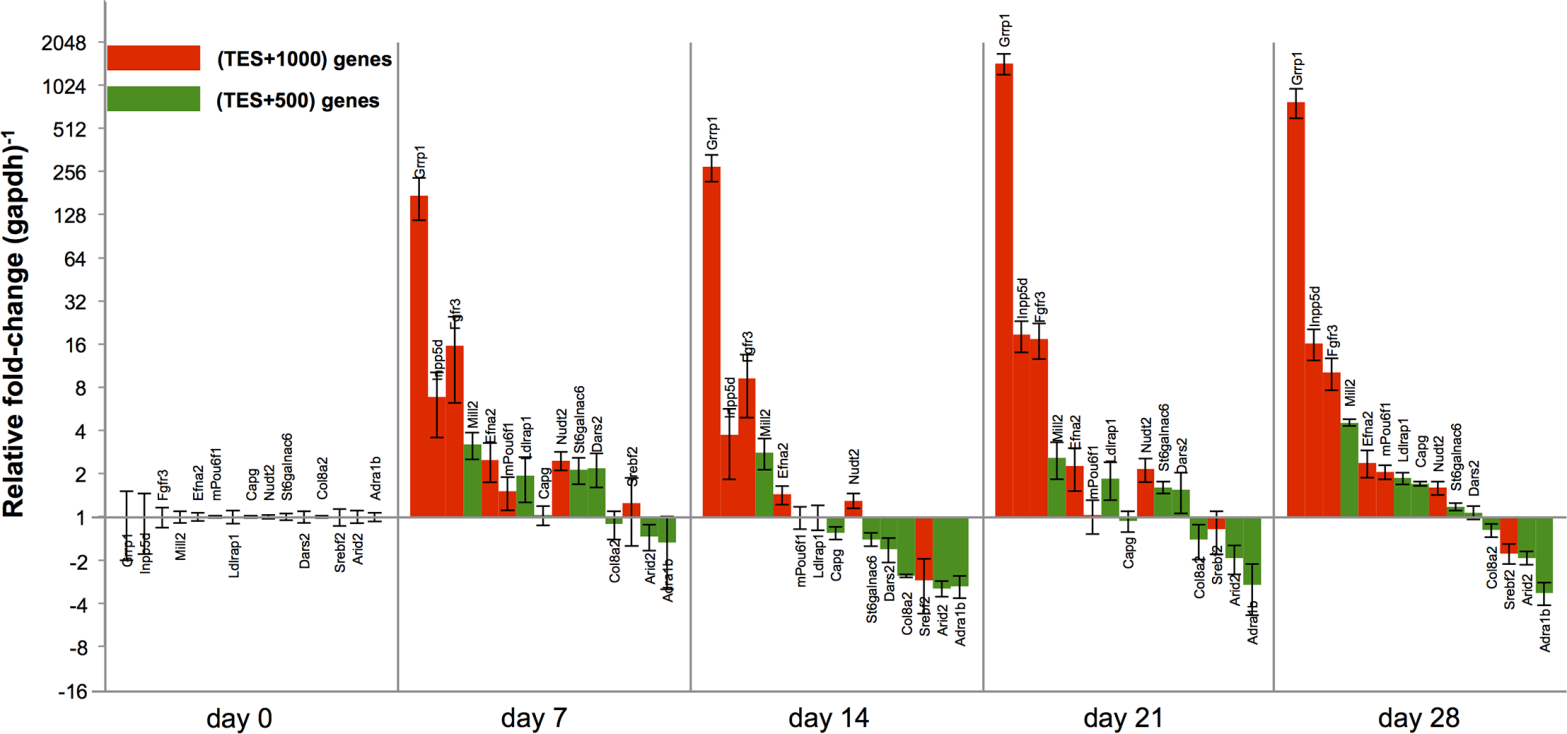
Relative-fold expression of selected genes throughout osteoblastogenesis. 15 genes displaying differential hypersensitivity at the TES were chosen from the TES+1000 (red) or TES+500 (green) gene list and subjected to RT-qPCR analysis at 7-day intervals throughout osteoblast differentiation of MC3T3-E1 cells (pre-osteoblasts, d7, d14, d21, and mineralizing osteoblasts). Relative-fold expression is represented as fold-change in message levels normalized to *gapdh* levels. Error bars represent ±SD.

We investigated whether enriched hypersensitivity at TES positions can be linked to differential expression. We selected 15 genes belonging to either the TES+1000 or TES+500 gene clusters that were not identified by the previous Affymetrix chip array to be differentially expressed during osteoblastogenesis (Table 1). These genes were then probed for changes in RNA levels throughout differentiation. Purified RNA from MC3T3-E1 cultures at days 0, 7, 14, 21, and 28 post-differentiation were collected and RT-qPCR analysis was performed (Fig 5). The profiles of these tested genes indeed showed changes in transcript levels during differentiation. Several genes are related to bone homeostasis, *Adra1b*, *Fgfr3*, *Col8a2*, *EfnaA2*, and *Ldlrap1*.

**Table 1:** Selected TES+1000 or TES+500 genes.

Gene name	Gene ID	Protein name	DHS cluster
Adra1b	NM_007416	adrenergic receptor, alpha 1b	TES+500
Arid2	NM_175251	AT rich interactive domain 2 (ARID, RFX-like); RIKEN cDNA 1700124K17 gene	TES+500
Capg	NM_007599	capping protein (actin filament), gelsolin-like	TES+500
Col8a2	NM_199473	collagen, type VIII, alpha 2	TES+500
Dars2	NM_172644	aspartyl-tRNA synthetase 2 (mitochondrial)	TES+500
Efna2	NM_007909	ephrin A2	TES+1000
Fgfr3	NM_008010	fibroblast growth factor receptor 3	TES+1000
Grrp1	NM_001099296	glycine/arginine rich protein 1	TES+1000
Inpp5d	NM_010566	inositol polyphosphate-5-phosphatase D	TES+1000
Ldlrap1	NM_145554	low density lipoprotein receptor adaptor protein 1	TES+500
Mill2	NM_153760	MHC I like leukocyte 2	TES+500
Nudt2	NM_025539	nudix (nucleoside diphosphate linked moiety X)-type motif 2	TES+1000
Pou6f1	NM_010127	POU domain, class 6, transcription factor 1	TES+1000
Srebf2	NM_033218	sterol regulatory element binding factor 2	TES+1000
St6galnac6	NM_016973	ST6 (alpha-N-acetyl-neuraminyl-2,3-beta-galactosyl-1,3)-N-acetylgalactosaminide alpha-2,6-sialyltransferase 6	TES+500

**Fig 6:**
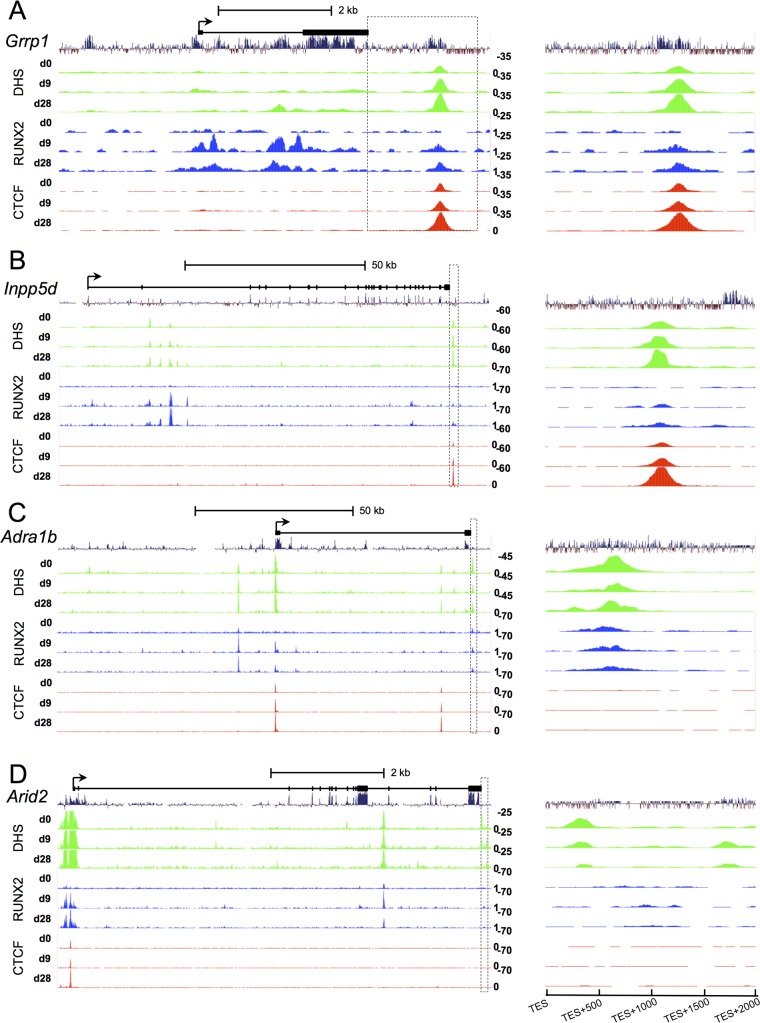
Signal tracks of select TES+1000 and TES+500 genes. *Grrp1* **(A),** and *Inpp5d* (**B**), *Adra1b* **(C),** and *Arid2* **(D)** show the relative signal intensities compared to RUNX2 enrichment (blue tracks) and CTCF enrichment (red tracks) encompassing entire gene bodies (left columns). Above each track is accompanied by 30-way Multiz Alignment conservation tracks as well as diagrams of the gene bodies. Boxed areas designate TES+2 kb windows that are also expanded in view (right columns) to highlight DHS peak centers at the 3’-ends. Peak summits for each gene are: *Grrp1*, TES+1280; *Inpp5d*, TES+1090; *Adra1b*, TES+650; and *Arid2*, TES+335.

We showed above that both RUNX2 and CTCF are prominently centered at many *cis*-regulatory regions. Coincidently, the two most upregulated TES+1000 genes: Glycine/arginine rich protein 1 (*Grrp1*) and Phosphatidylinositol-3,4,5-trisphosphate 5-phosphatase 1 (*Inpp5d*) (Fig 6A and 6B), and the two most downregulated TES+500 genes: *Adra1b* and AT-Rich Interactive Domain 2 (*Arid2*) (Fig 6C and 6D), exhibited increased RUNX2 and/or CTCF enrichments within their promoters or gene bodies. Coincidently, these four genes have significant roles in bone formation: Microarray analysis has shown that Runx2-/- embryo have a reduction of grrp1 expression in embryonal bone [53]; *Inpp5d* (SHIP1) is found in mesenchymal stroma osteoprogenitor cells [54]; *Adra1b* α1B -adrenoceptor signaling is required for bone formation and homeostasis [55], and ARID2 is a protein constituent of the SWI/SNF complex [56]. These correlations further suggest that DHS features at the TES+1000 and TES+500 may be used as indicators of gene expression change.

**Fig 7:**
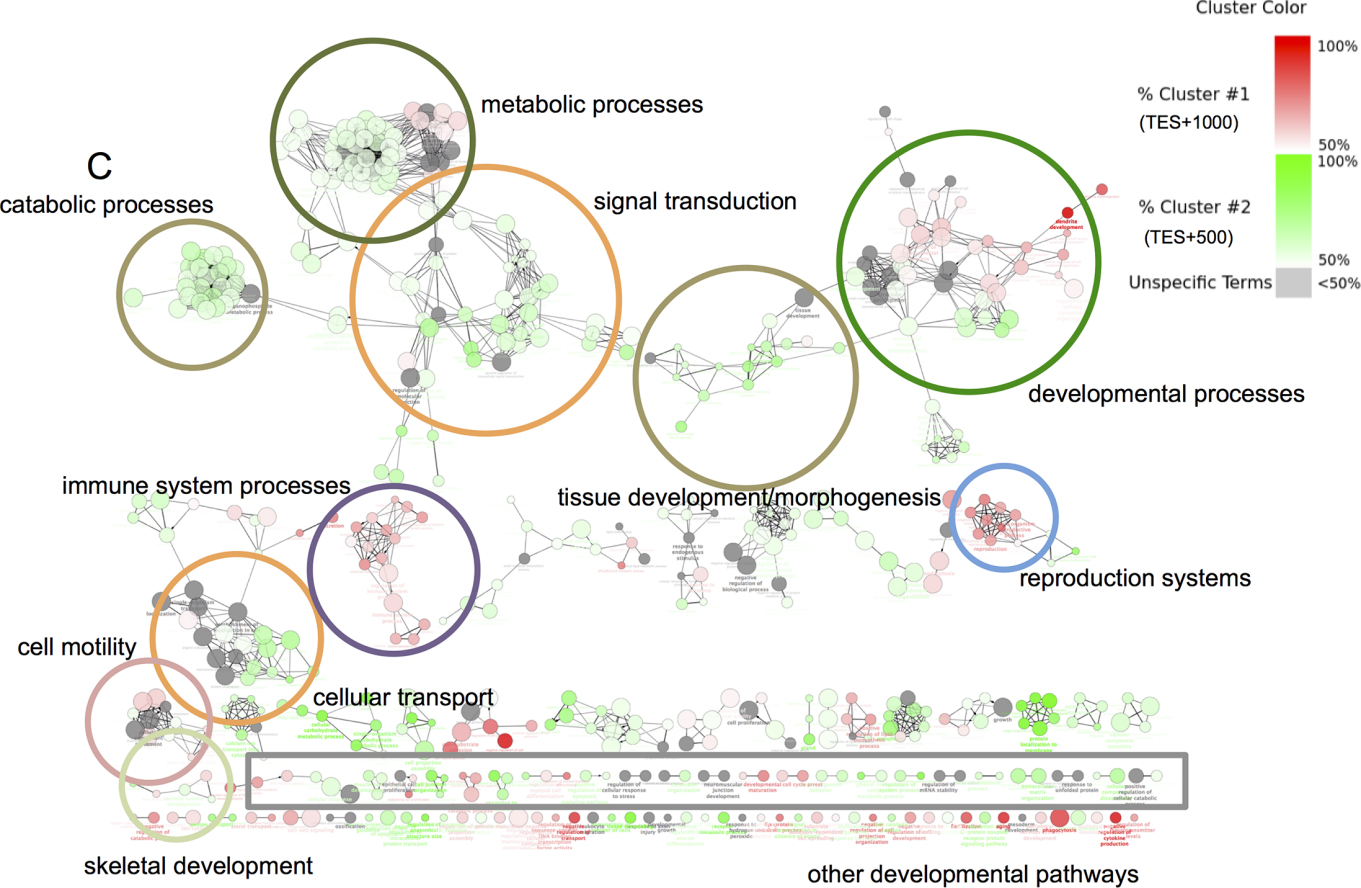
Genes with differentially hypersensitive 3’ regions near TES+1000 or TES+500 strongly correlate with developmental gene ontology pathways. Ontology term enrichment analysis of GO_Biological Processes was performed on the group of genes clustered by the presence of DHS peaks centered near TES+1000 or TES+500 sequences. Each node represents an enriched ontology term. Enrichment terms that were defined by 51% or more by TES+1000 genes are shaded in red, while terms defined by 51% or more by the TES+500 cluster of genes are shaded in green. Color intensities of each node reflect the relative strength they are enriched by either group. Gray nodes are terms equally enriched by both gene groups (Unspecified terms). The gray box represents ontology terms associated with various developmental pathways. Term nodes related to developmental processes, reproductive systems, and immune response processes are defined more by TES+1000 genes, while term nodes for metabolic and catabolic processes, signal transduction, cellular transport, and tissue or morphogenesis processes are defined more by the TES+500 genes.

Lastly, all genes at the DHS enriched TES regions were further interrogated for biologically relevant gene ontology terms related to osteoblastogenesis (Fig 7). Several of these terms are specifically related to cell developmental processes, morphogenesis, signal transduction pathways, and skeletal development. Enriched terms also related to general metabolic and catabolic processes. These findings support our conclusion that genes that are associated with the TES regions show expression trends that demonstrate correlation between differential 3’ hypersensitivity and gene expression.

In summary, our analyses demonstrate that the expression of bone-related genes are marked by DHS signals proximal to the TES. Furthermore, DHSs at the TES strongly implicates the 3’ region of genes as an additional parameter for identifying distinct subsets of regulated genes during osteoblastogenesis.

## Discussion

The findings of our global study support DNase hypersensitivity as a dynamic feature of gene regulation. Both changes in DHS enrichment and genomic location revealed among the three distinct subpopulations of osteoblastic cells have revealed several novel aspects of gene regulation during differentiation. Among these are: 1) a reduction of chromatin accessibility during osteoblast differentiation, reflecting a repression of genomic loci during differentiation; 2) the finding that DHS sites are highly dynamic at non-promoter regions, indicating the importance of regulatory mechanisms that take place beyond gene promoters; and 3) occupancy of RUNX2 and CTCF at intergenic and intronic DHSs further suggest that non-promoter DHS regions are key to establishing bone specificity; and 4) a unique pattern of DHSs at TES+500 and TES+1000 position may be indicators of gene expression change. We establish these 3’ flanking events as novel hallmarks for a specific class of genes that regulate commitment and osteoblast differentiation stages. Using this osteogenic model, we have demonstrated the potential for DNase hypersensitivity analysis to discover new elements of gene regulation. In conjunction with other high-throughput profiling methods, DNase-seq is considered a powerful tool for evaluating the mechanisms of gene regulation in differentiation systems, and can shed light into novel regulatory mechanisms that drive osteoblast differentiation [18]. Like other large-scale, high-throughput data studies that rely on peak calling algorithms to define changes in chromosomal states, our findings on their own are similarly limited. Specifically, genomic positions identified as stage-specific peaks in our analysis require further experimentation in future studies to reveal changes in functional activity between differential stages.

The observation that pre-osteoblasts are the most enriched overall in DHSs likely reflects the open chromatin mesenchymal precursor state. It has been shown that during embryonic stem cell differentiation, hypersensitivity diminishes throughout the genome [57]. The decrease in total DHSs in differentiated osteoblasts is perhaps indicative of silencing of non-osteogenic *cis*-regulatory regions that reinforces the osteoblast transcriptional profile during differentiation to mature bone. We therefore conclude that reduction in chromatin accessibility during osteoblast differentiation reflects the repression of genomic loci during differentiation. These observations infer that pre-osteoblasts also retain some characteristic of multipotency via a permissive chromatin landscape, and may provide a mechanistic explanation for the ability of MC3T3-E1 cultures to trans-differentiate to the adipocyte lineage [58, 59].

Although the strongest DHS sites are found at promoters and regions surrounding the TSS, the weaker but more abundant intergenic and intronic DHS sites are shown to be more dynamic. A recent study examining human derived MSC cells committed to the first stage of osteogenesis also found ~30% of DHS genomic domains were found in promoters. The majority of DHSs were found in other regions and many sites were unique to differentiated osteoblasts and associated with bone related genes [18]. In our study, we have discovered a novel category of regulatory control for a subset of genes regulated by 3’ flanking sequences. For example, *Dlx2* and *Sp7* are essential for osteogenesis commitment, yet they lack differential DHS signals at their promoters during differentiation. Instead, the DHS signals at their TESs are drastically increased. This change likely impacts their respective expression levels throughout commitment. Further exploration into the mechanism underpinning 3’ transcriptional regulation towards supporting a cell phenotype is needed. Notably, there are previous examples describing the looping of genes at the TSS to the TES during the transcriptional activation of genes [60, 61]. When interrogation of TES regions is coupled with other epigenetic profiling methodologies, it can provide a more comprehensive understanding of the chromatin landscape surrounding differentiation genes. Future in vivo DHS studies will also provide insights into how 3’ transcriptional regulation drives the process of embryogenesis.

Motif analyses of all DHS regions during osteoblast differentiation suggest that 12.1% of all DHSs were bound by RUNX2 and 9.4% by CTCF. This is in stark contrast to the 32.6% and 26.0% enrichments of RUNX2 and CTCF at DHSs by ChIP-seq analyses, respectively. The differences between motif and ChIP-seq analysis relate to the limitations of the *de novo* motif overrepresentation strategy. Nonetheless, bioinformatic-based motif analysis showed an unexpected preference in matrix depositing osteoblasts for RUNX2 binding to a motif containing a signal nucleotide difference at the third core base (5’-TG[C/T]GGTT-3’). This finding may indicate a unique mechanism for supporting the stability of the bone phenotype, perhaps by regulation through a higher affinity RUNX2 site.

By combining ChIP-seq, RNA-seq, and DNase-seq analysis, we have revealed a novel regulatory mechanism that supports osteoblast subpopulations as they reach terminal differentiation. For example, the finding that 10% of DHS sites share both RUNX2 and CTCF enrichment suggests potential cooperativity between these two factors. We note that both of these genes have known roles in the control of chromatin organization. To our knowledge, a role for CTCF in establishing the bone program has yet to be reported. In addition, CTCF was not identified in our Affymetrix data to be among genes that were differentially expressed. This cooperation expands upon their respective significance for commitment and differentiation as osteogenic cells undergo genome-wide regulatory change to synthesize the ECM and to initiate the mineral deposition process.

RUNX2 has long been known as the master factor of bone formation [38]. We now show quantitatively that RUNX2 is bound to a third of all *cis*-regulatory regions at the matrix deposition stage. Deletion of *Runx2* in mouse models causes an absence of bone formation in the developing skeleton with perinatal lethality, and various mutations in the human gene cause Cleidocranial Dysplasia [62, 63]. The high occurrence of RUNX2 enrichment at DHS-free regions remains intriguing. There are several possible explanations to this observation: RUNX2 can be enriched at these positions indirectly and bound to compacted chromatin as part of a larger scaffolding complex to recruit chromatin remodelers. For example, RUNX2-dependent chromatin remodeling during differentiation is reliant on BRG1, a subunit of the SWI/SNF complex [64], and RUNX2 is able to recruit the histone acetyl transferase p300 [65]. DHS sites within intergenic regions are considered to function as enhancer domains that can interact with promoter sequences far-distal from gene bodies via looping interactions. DHS sites are implicated in establishing intra- and even interchromosomal interactions [6]. These properties are consistent with earlier studies that demonstrate RUNX2 interacts with other factors to facilitate increased transcription of osteocalcin, e.g. by forming a loop between a distal VDR and the proximal TFII regulatory element [66]. Alternatively, RUNX2 may be enriched at these regions to silence or repress transcriptional modulation through co-regulatory proteins [67]. This suggestion is plausible, as RUNX2 is known as both a repressor and activator, depending on the co-regulatory partners it associates with [68]. A more speculative suggestion is that RUNX2 could act in these instances as a pioneering factor—i.e., binding to compacted chromatin to initiate accessibility at *cis*-regulatory regions. Many known master factors have been shown to have pioneering function, as FOXA1 and GATA transcription factors are among the first to engage silent genes, helping to endow competence for cell-type specification [69, 70]. Such a role has not been formally shown for RUNX2.

## Conclusion

These studies performed in a model for osteoblastogenesis have demonstrated that the use of global DNase hypersensitivity analysis in conjunction with ChIP-seq analysis of tissue-specific transcription factors can discover novel modes of gene regulation that are characteristic of subpopulations of cells at distinct stages during differentiation. Our studies are applicable to other differentiation models to provide a measurable index of global regulation by a master transcription factor of interest.

## Supporting information

S1 Table List of primer pairs used for qPCR analysis. (DOCX)

S2 Table Summary of the total number of mapped reads and F-seq called peaks from biological replicates 1 and 2 of pre-osteoblast (d0), matrix deposition (d9), and mineralization (d28) stages. (DOCX)

S1 Fig Osteoblast markers reflect expected kinetics of osteoblastogenesis throughout the 28-day differentiation timecourse. RT-qPCR analysis of three bone-related gene transcripts (*runx2P1*, blue line), (*ibsp*, red line), and (*bglap2*, green line) show message expression coincides with the osteoblastogenesis phenotype. Relative expression levels are represented as fold-change and normalized to *gapdh* on a log_2_ scale, n = 6. Error bars represent +1SD.
(TIF)

S2 Fig Violin plots demonstrating variable DHS lengths throughout differentiation. **(A)** Plots of DHS sites within pre-osteoblasts, matrix depositing osteoblasts, and mineralizing osteoblasts. Peaks were further subdivided into genomic partition categories: all peaks (gray), coding exons (black), all intronic sequences (dark green), first introns (light green), promoters (red), and intergenic sequences (blue). **(B)** DHS sites containing overlapping RUNX2 enrichment peaks. The y-axes represent the bp lengths of DHS sites. The x-axes represent the relative abundance of DHS sites of a particular length. As a whole, DHS sites were highly variable in length, but the majority ranged between ~300–500 bp as diagrams exhibited a “decanter” shape with the body centered at ~400 bp length. **(C)** Violin plots illustrating DHS length distributions between differentially gained DHS (inner solid plots) between pre-osteoblasts and matrix depositing osteoblasts (top graphs), pre-osteoblasts and mineralizing osteoblasts (middle graphs), and matrix depositing osteoblasts and mineralizing osteoblasts (bottom graphs), versus the lengths of all observed DHS sites (outer lines of plots) at either matrix depositing osteoblasts (top graphs), or mineralizing osteoblasts (middle and bottom graphs). The y-axes are the DHS lengths while the x-axes are the relative abundance of peaks at the DHS length. All differentially gained DHS sites (gray), coding exons (black), intronic sequence (dark green), first introns (light green), promoters (red), and intergenic sequences (blue) show that differential DHS sites are much shorter, ranging from ~200–300 bp in length, which is characteristic of enhancer regions that tend to be between ~100–500 bp in length. Many static DHS sites have lengths greater than or equal to 1 kb, which suggests that these DHS regions have a longer peak range whose positions may define large chromatin regions that are established by multi-protein complexes. **(D)** Violin plots illustrating DHS length differences of differentially gained DHS overlapping RUNX2 enrichment peaks (inner solid plots) between pre-osteoblasts and matrix depositing osteoblasts (top graphs), pre-osteoblasts and mineralizing osteoblasts (middle graphs) and matrix depositing osteoblasts and mineralizing osteoblasts (bottom graphs), versus the lengths of all observed DHS sites (outer lines of the plots) at either matrix depositing osteoblasts (top graphs), or mineralizing osteoblasts (middle and bottom graphs). Interestingly, DHS regions that span RUNX2 enrichment peaks are on average slightly larger (~400–600 bp)(compare S2A and S2B Fig,). This trend also holds true for differentially enriched DHS regions throughout all genic positions (compare S2C and S2D Fig). This result suggests that RUNX2-mediated transcription is centered at larger multi-complex regulatory regions, coinciding well with its known role as a nuclear scaffolding factor [21].
(TIF)

S3 Fig *De novo* discovery of motif enrichment among the three hallmark osteoblast stages. HOMER display outputs of the top 18 *de novo* discovered motifs enriched within DHS defined regions among **(A)** pre-osteoblast, **(B)** matrix deposition, and **(C)** mineralizing osteoblasts are shown. Motifs are ranked by P-value. The percentages that each motif is present within all DHS sites (% Targets) and within randomized sequences (% of Background). Each motif is designated a “best match” to a known factor binding consensus motif.
(TIF)
