# Supplementary material for: Genome-wide DNase hypersensitivity, and occupancy of RUNX2 and CTCF reveal a highly dynamic gene regulome during MC3T3 pre-osteoblast differentiation
Source: PLoS One. 2017 Nov 27;12(11):e0188056. doi: 10.1371/journal.pone.0188056 (PMC5703546; doi:10.1371/journal.pone.0188056)
Supplement: S1 Table — (DOCX) [file pone.0188056.s001.docx]

| mRNA target | Left Primer Sequence | Right Primer Sequence |
| --- | --- | --- |
| Adra1b | 5'-ACCTTGGGCATTGTAGTCGG-3' | 5'-GGGGCTTTAGGGTGGAGAAC-3' |
| Arid2 | 5'-ACCTACATGCAGAGTGTGGC-3' | 5'-TGGATCTGCATGCTGACTGG-3' |
| Capg | 5'-CTGGGGACTGCTTCATCCTG-3' | 5'-TGATGATTTCCACCTGGGCC-3' |
| Col8a2 | 5'-CTGGAATGAGCCGGCTCTAG-3' | 5'-AGAGAAGGCAGCCATCCCTA-3' |
| Dars2 | 5'-GCCAAGGAAGAGAGTCCCAC-3' | 5'-GTTGGCCACGAACCTTTTCC-3' |
| Efna2 | 5'-CAACCCCAGGTTTCAGGTGA-3' | 5'-GTCGTTGATGCTCACCTCCA-3' |
| Fgfr3 | 5'-AAGGTGTACAGCGATGCACA-3' | 5'-CGCCTGCAGTCTTGAGTACA-3' |
| Grrp1 | 5'-CCTTCTGCCTTCAGTCTGGG-3' | 5'-GGTCAGGGCTCCATACACAG-3' |
| Inpp5d | 5'-CCAAGAATGGTCCTGGCACT-3' | 5'-AGCAGCTTGAGTGGAACTCC-3' |
| Ldlrap1 | 5'-ACCTTGGTATGACGCTGGTG-3' | 5'-GAGTGTCACCTTCTGCAGCT-3' |
| Mill2 | 5'-GGGGAAATGAGACCTGTGCA-3' | 5'-GGAGAGTGTGAAGGCCTTCC-3' |
| Nudt2 | 5'-AGGCATCAGACGGCATTCAT-3' | 5'-TGATGGTCAGTTGGCTTGCT-3' |
| Pou6f1 | 5'-TTTAAGATCCGGCGGCTCTC-3' | 5'-GCTCTTGGGTGTGATGTCCA-3' |
| Srebf2 | 5'-CAAGTCAGCAGCCAAGGAGA-3' | 5'-CAAGGACTCCACCGCTCTTT-3' |
| St6galnac6 | 5'-CATGCAGCAGTTTGACGACC-3' | 5'-TCAGGAGGGACCATGCCATA-3' |

**Supplemental Table 1.** List of primer pairs used for qPCR analysis.
