# Supplementary material for: Genome-wide DNase hypersensitivity, and occupancy of RUNX2 and CTCF reveal a highly dynamic gene regulome during MC3T3 pre-osteoblast differentiation
Source: PLoS One. 2017 Nov 27;12(11):e0188056. doi: 10.1371/journal.pone.0188056 (PMC5703546; doi:10.1371/journal.pone.0188056)
Supplement: S2 Table — (DOCX) [file pone.0188056.s002.docx]

|  | **replicate** | **total mapped reads** | **F-seq called peaks** |
| --- | --- | --- | --- |
| **d0** | rep1 | 57,115,111 | 333,211 |
|  | rep2 | 22,559,212 | 177,923 |
| **d9** | rep1 | 24,857,081 | 217,734 |
|  | rep2 | 17,339,229 | 333,177 |
| **d28** | rep1 | 35,905,988 | 283,329 |
|  | rep2 | 29,740,309 | 211,325 |

**Supplemental Table 2.** Summary of the total number of mapped reads and F-seq called peaks from biological replicates 1 and 2 of pre-osteoblast (d0), matrix deposition (d9), and mineralization (d28) stages.
