# Supplementary figures and images for: Genome-wide DNase hypersensitivity, and occupancy of RUNX2 and CTCF reveal a highly dynamic gene regulome during MC3T3 pre-osteoblast differentiation
Source: PLoS One. 2017 Nov 27;12(11):e0188056. doi: 10.1371/journal.pone.0188056 (PMC5703546; doi:10.1371/journal.pone.0188056)

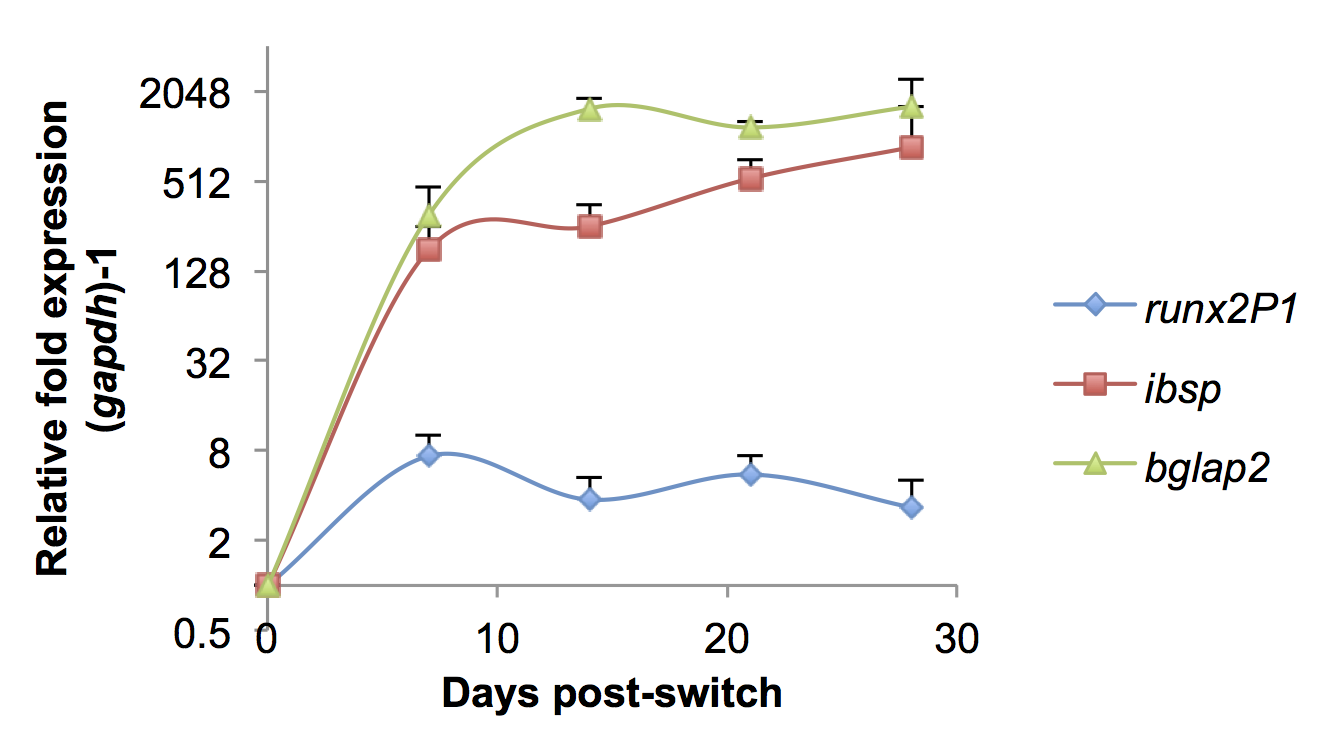

Supplement: S1 Fig — RT-qPCR analysis of three bone-related gene transcripts (runx2P1, blue line), (ibsp, red line), and (bglap2, green line) show message expression coincides with the osteoblastogenesis phenotype. Relative expression levels are represented as fold-change and normalized to gapdh on a log2 scale, n = 6. Error bars represent +1SD. (TIF) [file pone.0188056.s003.tif]

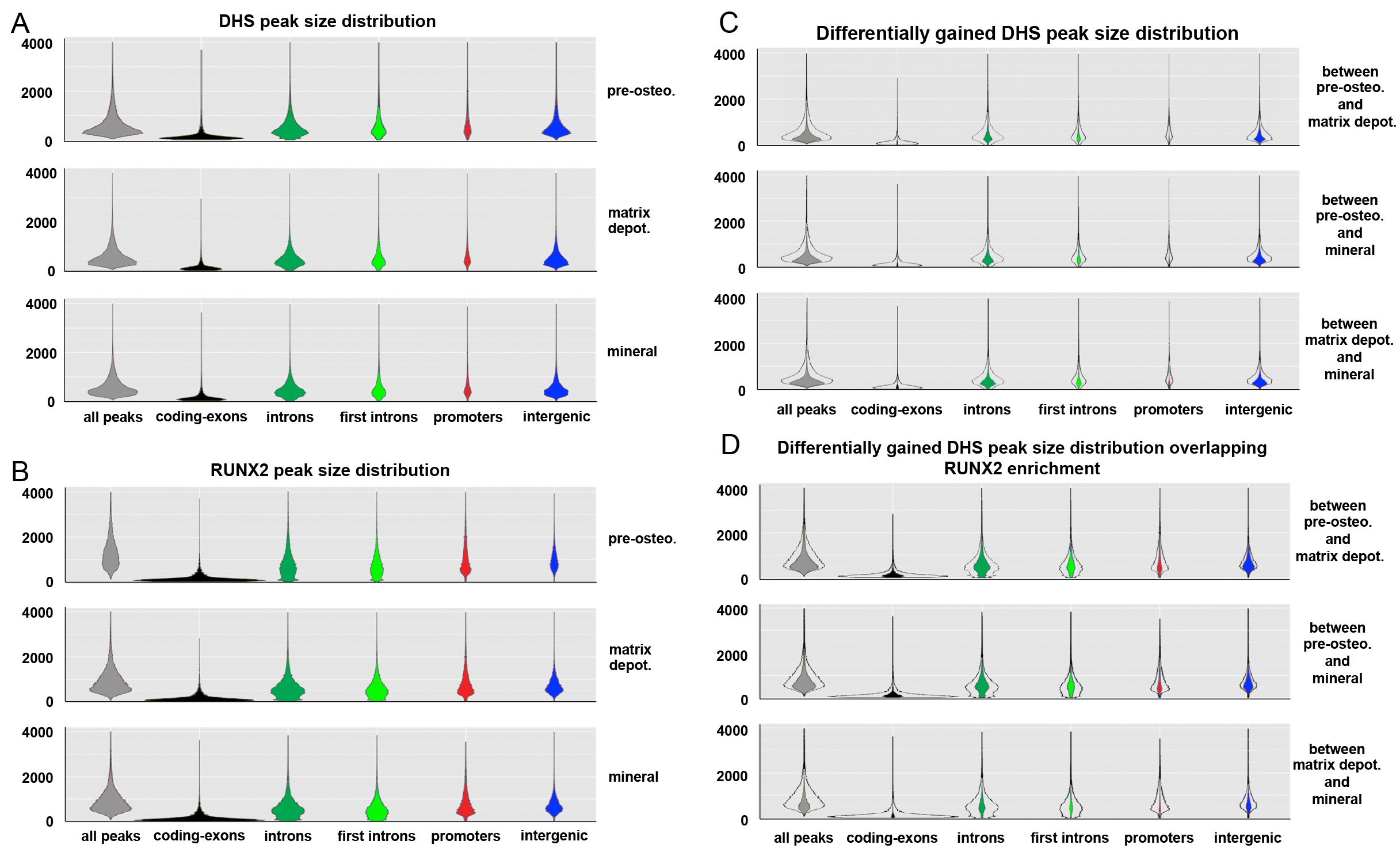

Supplement: S2 Fig — (A) Plots of DHS sites within pre-osteoblasts, matrix depositing osteoblasts, and mineralizing osteoblasts. Peaks were further subdivided into genomic partition categories: all peaks (gray), coding exons (black), all intronic sequences (dark green), first introns (light green), promoters (red), and intergenic sequences (blue). (B) DHS sites containing overlapping RUNX2 enrichment peaks. The y-axes represent the bp lengths of DHS sites. The x-axes represent the relative abundance of DHS sites of a particular length. As a whole, DHS sites were highly variable in length, but the majority ranged between ~300–500 bp as diagrams exhibited a “decanter” shape with the body centered at ~400 bp length. (C) Violin plots illustrating DHS length distributions between differentially gained DHS (inner solid plots) between pre-osteoblasts and matrix depositing osteoblasts (top graphs), pre-osteoblasts and mineralizing osteoblasts (middle graphs), and matrix depositing osteoblasts and mineralizing osteoblasts (bottom graphs), versus the lengths of all observed DHS sites (outer lines of plots) at either matrix depositing osteoblasts (top graphs), or mineralizing osteoblasts (middle and bottom graphs). The y-axes are the DHS lengths while the x-axes are the relative abundance of peaks at the DHS length. All differentially gained DHS sites (gray), coding exons (black), intronic sequence (dark green), first introns (light green), promoters (red), and intergenic sequences (blue) show that differential DHS sites are much shorter, ranging from ~200–300 bp in length, which is characteristic of enhancer regions that tend to be between ~100–500 bp in length. Many static DHS sites have lengths greater than or equal to 1 kb, which suggests that these DHS regions have a longer peak range whose positions may define large chromatin regions that are established by multi-protein complexes. (D) Violin plots illustrating DHS length differences of differentially gained DHS overlapping RUNX2 enrichment [file pone.0188056.s004.tif]

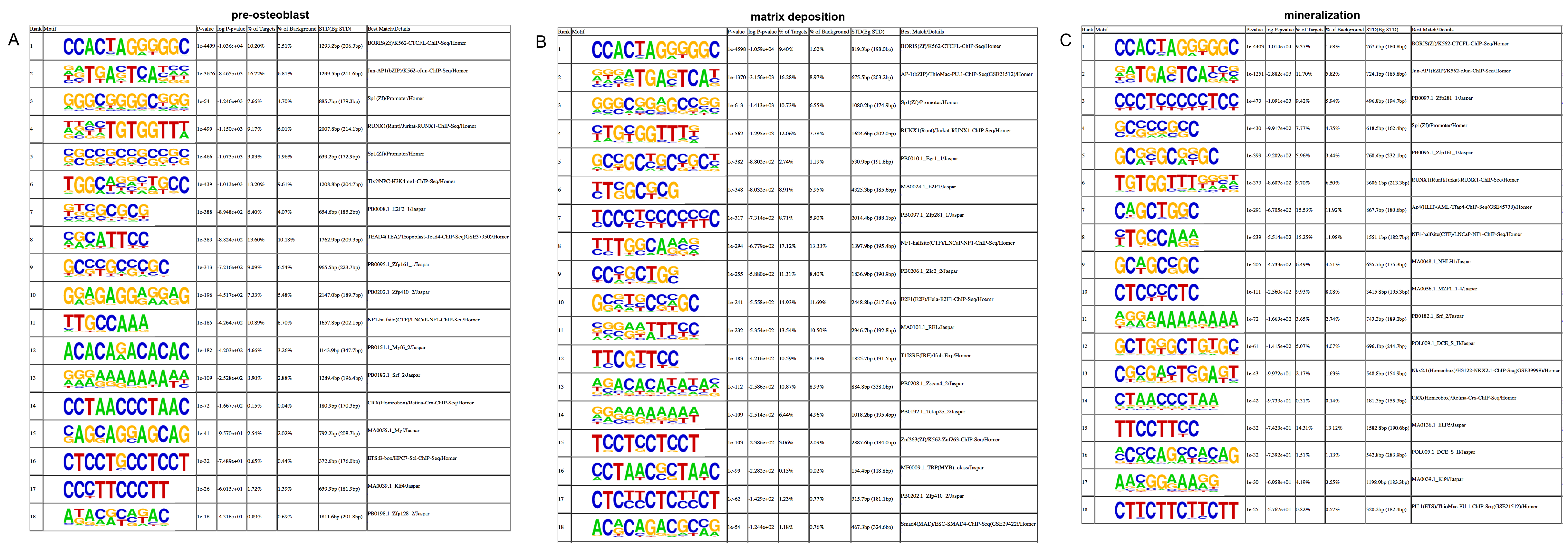

Supplement: S3 Fig — HOMER display outputs of the top 18 de novo discovered motifs enriched within DHS defined regions among (A) pre-osteoblast, (B) matrix deposition, and (C) mineralizing osteoblasts are shown. Motifs are ranked by P-value. The percentages that each motif is present within all DHS sites (% Targets) and within randomized sequences (% of Background). Each motif is designated a “best match” to a known factor binding consensus motif. (TIF) [file pone.0188056.s005.tif]
